# Synthetic nanobodies as tools to distinguish IgG Fc glycoforms

**DOI:** 10.1073/pnas.2212658119

**Published:** 2022-11-21

**Authors:** Kevin S. Kao, Aaron Gupta, Guanghui Zong, Chao Li, Isabell Kerschbaumer, Sara Borghi, Jacqueline M. Achkar, Stylianos Bournazos, Lai-Xi Wang, Jeffrey V. Ravetch

**Affiliations:** ^a^Laboratory of Molecular Genetics & Immunology, The Rockefeller University, New York, NY 10065; ^b^Department of Chemistry and Biochemistry, University of Maryland, College Park, MD 20742; ^c^Department of Medicine (Division of Infectious Diseases), Albert Einstein College of Medicine and Montefiore Medical Center, Bronx, NY 10461; ^d^Department of Microbiology and Immunology, Albert Einstein College of Medicine and Montefiore Medical Center, Bronx, NY 10461

**Keywords:** nanobody, glycobiology, immunoglobulin

## Abstract

Protein glycosylation is a crucial mediator of biological functions and is tightly regulated in health and disease. However, interrogating complex protein glycoforms is challenging, as current lectin tools are limited by cross-reactivity while mass spectrometry typically requires biochemical purification and isolation of the target protein. Here, we describe a method to identify and characterize a class of nanobodies that can distinguish glycoforms without reactivity to off-target glycoproteins or glycans. We apply this technology to immunoglobulin G (IgG) Fc glycoforms and define nanobodies that specifically recognize either IgG lacking its core-fucose or IgG bearing terminal sialic acid residues. By adapting these tools to standard biochemical methods, we can clinically stratify dengue virus and SARS-CoV-2 infected individuals based on their IgG glycan profile, selectively disrupt IgG–Fcγ receptor binding both in vitro and in vivo, and interrogate the B cell receptor (BCR) glycan structure on living cells. Ultimately, we provide a strategy for the development of reagents to identify and manipulate IgG Fc glycoforms.

## Significance

On proteins, glycans are attached through stepwise construction of linear or branched structures, ultimately leading to families of highly related but nonequivalent glycoproteins known as glycoforms. On immunoglobulin G (IgG), differences in glycosylation of the fragment crystallizable domain (Fc) modulate its ability to signal to leukocytes to execute critical antibody effector functions. Despite burgeoning interest in understanding the complexities of IgG Fc glycoforms, there is an evident scarcity of tools available to study them. Here, we identify nanobodies which we use to study and manipulate specific IgG glycoforms in vitro and in vivo.

Glycosylation is one of the most common post-translational modifications and is a critical modulator of biological processes. Many proteins can adopt a wide array of glycosylation states—referred to as glycoforms—which can have varying composition, structures, and physiological functions. Despite the importance of protein glycoforms, there is a scarcity of tools to study them. Over the years, there have been numerous attempts to generate glycan-binding reagents, such as lectins or antibodies (1–4). However, the majority are suboptimal due to cross-reactivity, poor affinity, and/or promiscuity for multiple glycoproteins. Furthermore, antibodies successfully targeting glycan epitopes are typically only specific for the carbohydrate motif but nonspecific for the particular glycoprotein displaying that motif (5–7).

At present, the most accurate and comprehensive method for studying protein glycosylation is mass spectrometry in conjunction with high-performance liquid chromatography (LC–MS) (8–10). Though additional methods, such as capillary electrophoresis or lectin arrays, are sometimes used (11–13), they also present methodological barriers that limit adaptability to molecular biology techniques such as enzyme-linked immunosorbent assay (ELISA) and flow cytometry and do not allow for in vivo manipulation of glycoproteins. This necessitates an alternative approach.

**Fig. 1.**
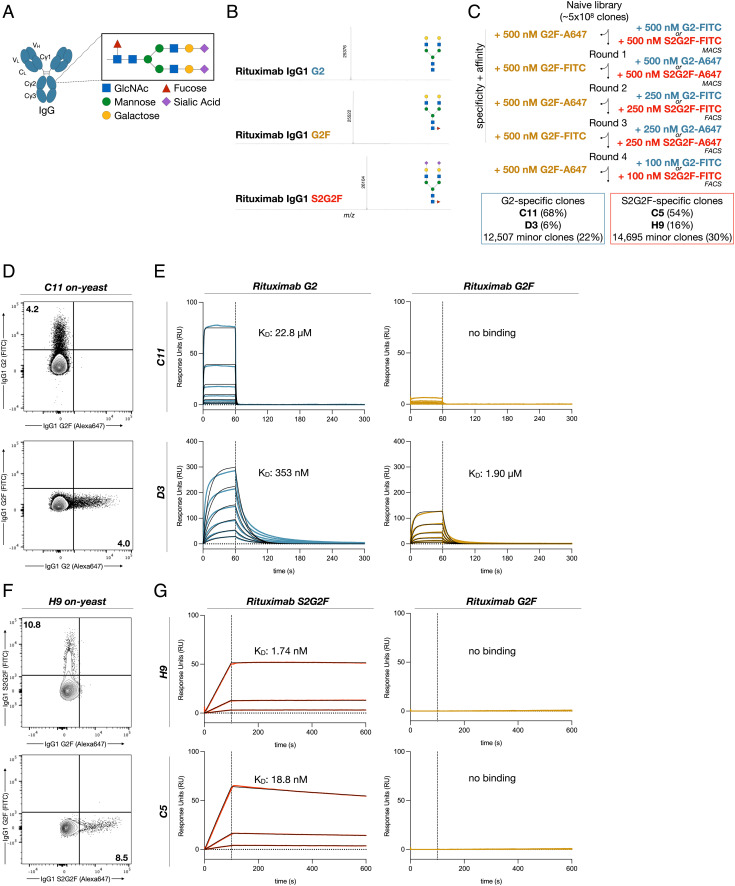
Generation of IgG glycoform-specific nanobodies. (*A*) Schematic of the N-linked glycan on Asn-297 of the IgG Fc. (*B*) Liquid chromatography electrospray ionization mass-spectrometry (LC–ESI–MS) of the G2 and G2F glycoforms of rituximab. G2-Fc, M = 25,377 Da; found (m/z) 25,376 (deconvolution data), G2F-Fc, M = 25,523 Da; found (m/z) 25,522 (deconvolution data), S2G2F-Fc, M = 26,105 Da; found (m/z) 26,104. (*C*) Selection strategy for identification of G2 or S2G2F glycoform-specific nanobodies via magnetic-activated cell sorting (MACS) or fluorescence-activated cell sorting (FACS). Library diversity following five rounds of selection was assessed by next generation sequencing. (*D*) Flow cytometry of yeast displaying C11 with fluorescently labeled IgG1 G2 and G2F glycoforms. (*E*) Binding kinetics of the two dominant clones specific for the G2 glycoform of IgG1 Fc, C11, and D3 evaluated by SPR. Blue or yellow traces are raw data, while 1:1 Langmuir global kinetic fits are shown in black. Sample concentrations began at 1024 nM with two-fold serial titration until 32 nM. (*F*) Flow cytometry of yeast displaying H9 with fluorescently labeled IgG1 G2F and S2G2F glycoforms. (*G*) Binding kinetics of the two dominant clones specific for IgG1 Fc S2G2F, C5, and H9. Blue or yellow traces are raw data, while global kinetic fits are shown in black. Sample concentrations began at 256 nM with four-fold serial titration until 16 nM.

To address this problem, we chose to target one of the most abundant glycoproteins in human serum, immunoglobulin G (IgG). This was an attractive target as all four subclasses of IgG possess a single complex, biantennary N-linked glycan on Asn297 (Fig. 1*A*). The presence of this glycan allows for 36 theoretical glycoforms, of which over 30 have been observed by mass spectrometry (14). These glycoforms have varying affinity and selectivity for Fcγ receptor (FcγR) binding (15, 16), thereby dictating their protective or pathogenic activity (17). More specifically, IgG lacking its core fucose residue has ~10–20-fold higher affinity for the activating FcγRIIIA (18), while terminal sialylation allows for engagement of Type II FcRs (19, 20). Though it is well established that IgG Fc glycan modifications are dynamically regulated both in health and disease, recent reports have provided support for the role of these modifications as prognostic indicators of disease progression in viral illness (21, 22). In dengue virus-positive patients, levels of afucosylated IgG1 antibodies at admission predict whether a patient will progress to severe disease, namely, dengue hemorrhagic fever (DHF) or dengue shock syndrome (DSS) (23). This same modification also stratifies and serves as a prognostic indicator of clinical severity in PCR-positive COVID-19 patients (24, 25).

Further, because the abundance of afucosylated IgG has predictive power in dengue virus and SARS-CoV-2 infection, a probe for this glycoform would open the door for rapid point-of-care tools that could be used to stratify patient risk based on disease-related changes to the IgG glycome. In addition, recent studies have suggested that afucosylated IgG glycoforms may enhance pathogenesis in some viral illnesses, indicating a potential avenue for therapeutics that target these complex structures. Finally, no methods to date can interrogate IgG glycosylation of the membrane-bound B cell receptor (BCR) of living cells, and thus, they cannot be used to study cellular regulation of this essential post-translational modification.

Nanobodies are used as therapeutic agents and diagnostic probes due to their small size, ease of production, and excellent specificity and affinity (26–28). Derived from camelid species, they share a similar molecular architecture with human and mouse immunoglobulin variable-heavy chain (V_H_) domains, with four conserved framework regions flanking three hypervariable complementarity determining regions (CDRs). However, the CDR3 in most camelids is substantially longer than that of mouse or human variable regions, enabling greater structural flexibility for recognition of recessed or otherwise inaccessible epitopes (29), as may be the case with the N-linked IgG glycan. To capitalize on these advantages and circumvent the challenges of animal immunization, we utilized a purely synthetic yeast nanobody display library that approximates camelid nanobody diversity in vitro (30). Further, because proteins produced by standard recombinant methods generally exist as a heterogeneous pool of glycoforms (31, 32), screening for glycoform-specific antibodies has previously been difficult and largely unsuccessful. To overcome this limitation, we chemoenzymatically glycoengineered IgG to adopt a single glycoform of interest, which we hypothesized would allow for selection of nanobodies with high degrees of specificity.

Using this approach, we successfully identified IgG glycoform-specific nanobodies. These molecules demonstrate exquisite specificity for both the complex, biantennary N-linked glycan as well as the protein backbone of IgG Fc. One such nanobody recognizing afucosylated IgG, B7, and its affinity matured progeny was adapted to standard biochemical assays such as ELISA, Luminex, and flow cytometry. This allowed for rapid quantification of afucosylated IgG in patient sera. In addition, we utilized higher affinity variants to selectively disrupt interactions between FcγRs and specific IgG glycoforms both in vitro and in vivo. Finally, we demonstrate specific nanobody binding to afucosylated BCR on both a lymphoblastic cell line and primary human B cells. These findings constitute the first discovery of broadly applicable tools that can distinguish complex protein glycoforms and provide a rational approach for the generation of additional glycoform-specific reagents.

## Results

### Discovery and Characterization of IgG Glycoform-Specific Nanobodies.

To precisely select for nanobodies specific for afucosylated and sialylated IgG, we chemoenzymatically engineered clinical grade rituximab into its galactosylated afucosylated (G2), galactosylated fucosylated (G2F), or galactosylated sialylated fucosylated (S2G2F) glycoforms (Fig. 1*B* and *SI Appendix*, Fig. S1), as previously described (33, 34). The identity and homogeneity of the glycoengineered glycoforms of rituximab were confirmed by liquid chromatography electrospray ionization mass spectrometry (LC–ESI–MS) analysis of the Fc domains released by IdeS treatment of the respective rituximab glycoforms. (*SI Appendix*, Fig. S1) While commercial rituximab consisted of three major Fc glycoforms, glycoengineered rituximab showed a single peak. Furthermore, for quantitative analysis, the Fc N-glycans were released from the antibodies, fluorescently labeled with 2-aminobenzoic acid (2-AA) and analyzed by high performance liquid chromatography (HPLC). The HPLC separation and quantification indicated that commercial rituximab carried three different N-glycans, G2F, G1F, and G0F, respectively, in a ratio of 9.3:47.7:43.0. However, the glycoengineered glycoforms carried clearly only the expected single Fc N-glycan without detection of other potential contaminant N-glycans (*SI Appendix*, Fig. S2). These results confirm the purity of the glycoengineered rituximab glycoforms. The three glycoforms were fluorescently labeled with FITC and Alexa Fluor 647 and yeast displaying nanobodies with specific affinity for the G2 or S2G2F glycoforms were selected through two rounds of magnetic-activated cell sorting (MACS) and three rounds of  fluorescence-activated cell sorting (FACS) (Fig. 1*C*). High-affinity clones were obtained by successively lowering the target glycoform concentration, while specificity was maintained throughout each round by counter-selecting against a high fixed concentration of the undesirable G2F glycoform. After the final round of selection, the resulting library was sequenced and single yeast clones were characterized by flow cytometry (Fig. 1 *D* and *F*). This screening strategy yielded two nanobodies specific for the G2 glycoform (C11, D3) and two nanobodies specific for the S2G2F glycoform (C5, H9) (Fig. 1 *D*–*G*). Although D3 bound the G2 glycoform with higher affinity than C11 (K_D_ = 323 nM vs. 22.8 µM), affinity for the G2F glycoform was demonstrably higher (K_D_ = 1.9 µM vs. n.b.). Because glycan binding reagents have typically suffered from poor affinity, we proceeded to mature C11. Sialylated IgG-specific clones C5 and H9 had sufficiently high affinities (K_D_ = 1.74 nM and 18.8 nM) and did not require further improvement (Fig. 1 *F* and *G* and *SI Appendix*, Fig. S3 *A*–*D*).

### Affinity Maturation and Multimerization of Nanobody Clones.

**Fig. 2.**
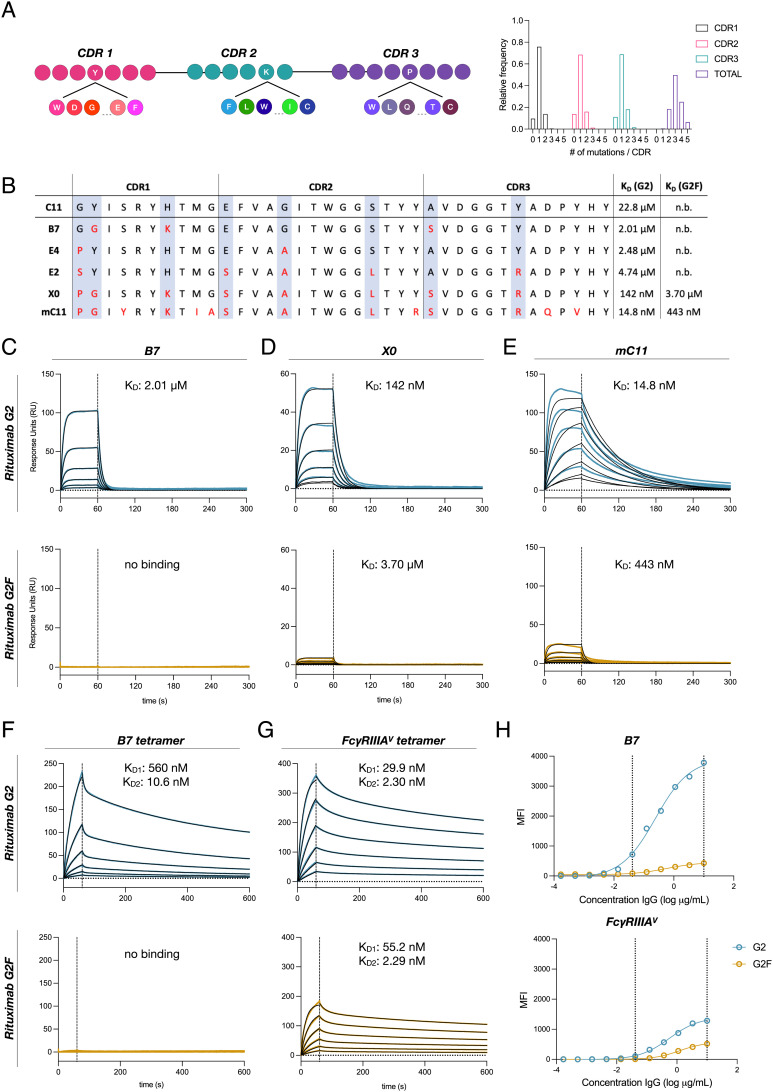
Affinity maturation of C11 yields nanobodies with nanomolar affinity. (*A*) Schematic representation of NNK site-saturation mutagenesis of C11’s three CDRs. High-throughput sequencing of resulting affinity maturation library demonstrates ~1 mutation per CDR for a total of ~3 per clone. (*B*) CDR sequences and dissociation constants (K_D_) for the G2 and G2F glycoforms for five afucosylation-specific high affinity clones. (*C–G*) Binding kinetics of B7, X0, mC11, tetrameric B7, or tetrameric FcγRIIIA with G2 or G2F glycoforms of rituximab evaluated by SPR. Blue or yellow traces are raw data, and kinetic fits are shown in black. Sample concentrations began at 256 nM with two-fold serial titration until 8 nM (*H*) Luminex assay comparing the specificity and sensitivity of tetrameric B7 with tetrameric FcγRIIIA for detecting the G2 or G2F glycoforms of rituximab. Vertical dashed lines indicate the range where G2 and G2F can be adequately distinguished. Data were fitted by nonlinear regression analysis.

To further affinity mature clones specific for afucosylated IgG, we designed a site-saturation mutagenesis library of the CDRs of C11 (Fig. 2*A*). Two rounds of selection of the resulting library, in which G2F was maintained in 50-fold molar excess of G2 bait, yielded numerous clones with penetrant mutations at specific ‘hotspots’ within each CDR. These clones demonstrated 10–1,000-fold affinity for G2 while retaining similar levels of specificity as C11 (Fig. 2 *B*–*E*). Combinatorial assembly of the mutations present in the top clones resulted in a dominant clone, mC11, which exhibited a 1,000-fold improvement in affinity for G2 when compared to the C11 parental clone at the cost of marginal specificity (Fig. 2*E*). Based on its exquisite specificity, we chose to focus on clone B7 and further engineer it for increased affinity. Nanobody multimers have been shown to possess drastically higher binding affinities, largely through avidity (35, 36). To take advantage of this property, we generated biotin-streptavidin tetramers of the most specific nanobody clone, B7. As expected, tetramerization greatly enhanced binding affinity for G2 (K_D1_ = 560 nM, K_D2_ = 10.6 nM), while preserving specificity (Fig. 2*F*).

Though some have proposed the use of soluble FcγRIIIA as a detection reagent for afucosylated IgG due to its higher affinity for these glycoforms (37), B7 tetramers demonstrated much greater specificity by surface plasmon resonance (SPR) as well as greater sensitivity in immunoassays (Fig. 2 *G* and *H*), demonstrating the advantages of the nanobody approach.

### IgG Glycoform-Specific Nanobodies Depend on Both Protein Backbone and Glycan Composition for Binding.

Antibodies and lectins specific for glycan residues are ubiquitous in research. However, to the best of our knowledge, reagents for specific complex protein glycoforms have not been reported. To rule out binding to free glycans, we probed an N-linked glycan array using B7. As expected, B7 only recognized the human IgG positive control and did not bind any of the N-glycans, regardless of fucosylation (*SI Appendix*, Fig. S4 *A* and *B*). The specificity of our glycan array was confirmed using the fucose-binding lectin Aleuria Aurantia Lectin (AAL). Similarly, we confirmed a lack of cross-reactivity to aglycosylated protein as B7 did not bind IgG1 N297A (*SI Appendix*, Fig. S5 *A* and *B*).

Human IgG is composed of four subclasses—IgG1, IgG2, IgG3, and IgG4—which share over 90% homology within their Fc domain. To test the subclass cross-reactivity of afucosylation-specific clone B7, we used G2 and G2F glycoforms formatted with human IgG1-4 Fc domains (38). B7 exhibited subclass specificity (IgG1 > IgG2 > IgG3 >> IgG4) (*SI Appendix*, Fig. S6 *A* and *B*) but surprisingly maintained specificity for afucosylated glycoforms, with the largest fold-change in specificity for IgG1 and IgG2. In contrast to IgG1, specific glycoforms of other subclasses have a limited biological role in disease, either due to their low abundance in serum or weak FcγR binding. Furthermore, only afucosylated IgG1 has been correlated with the clinical course of inflammatory diseases, while analysis of afucosylated glycoforms of IgG2-4 has demonstrated insignificant predictive power (21).

Finally, we verified that B7 retains binding to all afucosylated forms of IgG1 (G0, G2, and S2G2) regardless of galactosylation or sialylation (*SI Appendix*, Fig. S6*C*). Taken together, these studies demonstrate the strict requirements for both peptide sequence and glycan structures necessary for glycoform-specific nanobody binding.

### Afucosylated IgG-Specific Nanobodies Block IgG–FcγR Interactions In vitro and In vivo.

**Fig. 3.**
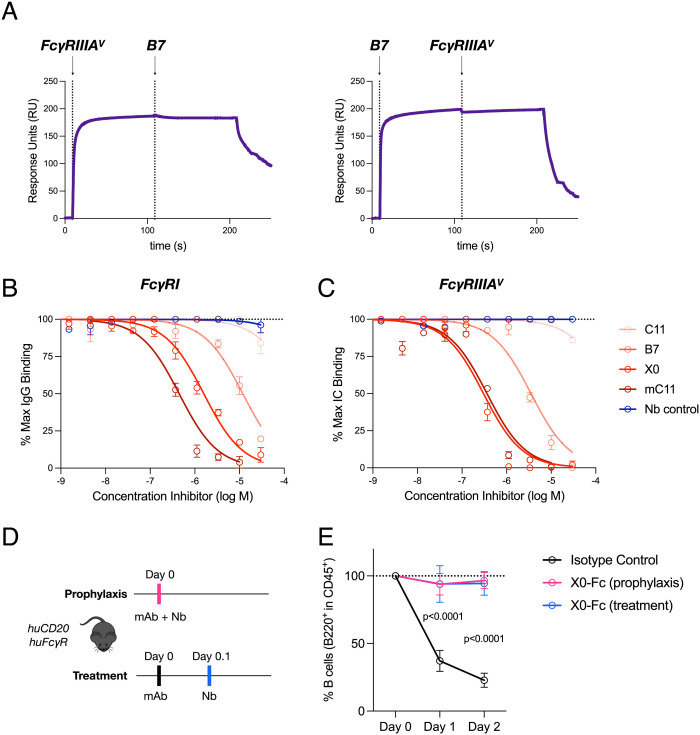
Afucosylated IgG-specific nanobodies block Fc–FcγR interactions in vitro and in vivo. (*A*) Epitope mapping by SPR shows mutually exclusive binding of B7 and FcγRIIIA to afucosylated IgG1. Vertical dashed lines indicate primary and secondary injection times. (*B* and *C*) ELISA evaluating nanobody inhibition of FcγRI or FcγRIIIA binding to afucosylated IgG or immune complexes, respectively. Data displayed as mean ± SEM. Data were fitted by nonlinear regression analysis. (*D* and *E*) Mice were coadministered X0-Fc (2.5 mg/kg) and rituximab G2 (0.5 mg/kg) either prophylactically or as treatment. Data displayed as mean ± SEM (n = 3–4 mice per group).

To better understand the impact of nanobody binding on IgG–FcγR interactions, we performed epitope mapping studies. These studies revealed mutually exclusive binding of B7 and FcγRIIIA to afucosylated IgG1. (Fig. 3*A*). Similarly, B7 and its higher affinity variants, X0 and mC11, competitively inhibited monomeric IgG or preformed immune complexes from binding multiple FcγR family members (Fig. 3 *B* and *C*).

Afucosylated IgG has been suggested to be a key pathological driver in severe cases of dengue virus and SARS-CoV-2 infection. The mechanisms of this disease enhancement purportedly rely on specific IgG–FcγR interactions. Given our preliminary data demonstrating the capacity for nanobody-mediated blockade, we explored whether clone X0, with intermediate affinity and high specificity, could be used in vivo as a therapeutic to prevent rituximab-mediated B cell depletion. We chose this model because our group and others have previously shown that only afucosylated rituximab is capable of depleting B cells in humanized FcγR mouse models (34). To ensure adequate serum half-life of our nanobody therapeutics, we generated nanobody-Fc fusions. Mice were either administered X0-Fc prophylactically or as a treatment following afucosylated rituximab. In both cases, X0-Fc completely blocked B cell depletion compared to isotype controls (Fig. 3 *D* and *E*). These studies collectively demonstrate that our glycoform specific nanobodies are potential therapeutics that can selectively target and manipulate specific protein glycoforms.

### Afucosylated IgG-Specific Nanobodies Can be Adapted to Prognostic and Diagnostic Assays for Severe Viral Infection.

**Fig. 4.**
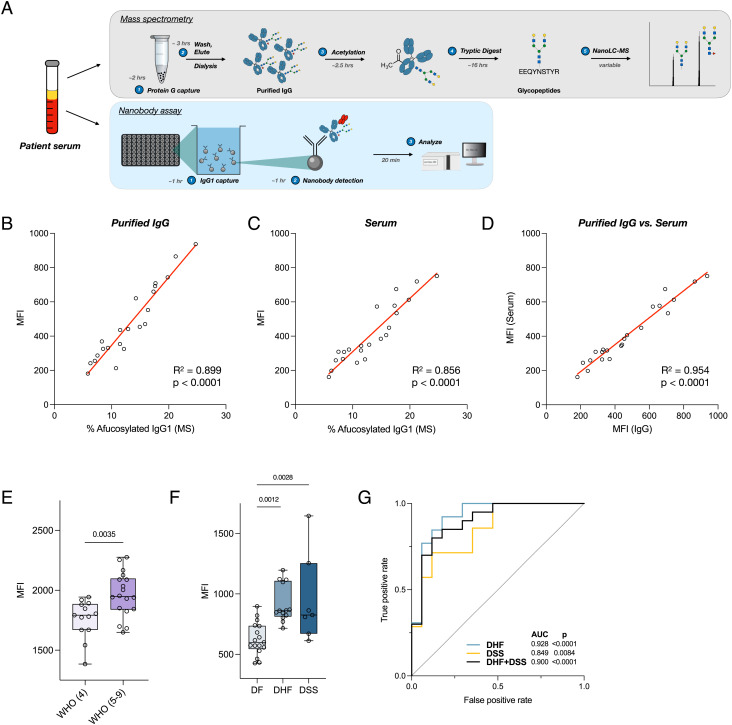
Nanobody tetramers allow for quantification of IgG glycan composition in patient samples. (*A*) Comparison of assay procedures between mass spectrometry and nanobody-based methods of IgG Fc glycan analysis. The nanobody-based assay is simply completed in under 3 h without the need for IgG purification or complex instrumentation. (*B* and *C*) Luminex assay quantifying afucosylated IgG1 levels in purified IgG or patient serum. (*D*) Correlation of afucosylated IgG1 levels detected in purified IgG versus patient serum. (*E*) Levels of afucosylated IgG1 in SARS-CoV2-infected hospitalized patients admitted with signs and symptoms of COVID-19, comparing patients with moderate disease not requiring supplemental oxygen (WHO score 4; n = 13) to those with moderate-to-severe disease requiring supplemental oxygen (WHO scores 5–9; n = 18). (*F*) Levels of afucosylated IgG1 in dengue patients with variable disease severity (dengue fever (DF), DHF, or DSS. Data displayed as box plot with all points plotted. Whiskers represent min and max. (*G*) Receiver operating characteristic (ROC) analysis for the predictive value of afucosylated IgG1 levels at hospital admission for progression to severe dengue infection. AUC, area under curve. Pearson correlation analysis for (*B*–*D*); One-way ANOVA/Bonferroni post hoc for (*E* and *F*). Boxes and whiskers represent the median, quartiles, and range (minimum to maximum); numbers above the boxes indicate *P* values.

Certain protein glycoforms can serve as powerful markers of specific disease states. Prior reports have demonstrated that the level of afucosylated IgG1 is a robust prognostic marker for severe dengue virus infection. A high level in newly admitted patients predicts disease progression to life-threatening  DHF or DSS (21). These studies have largely relied on low-throughput mass spectrometry methods to characterize levels of afucosylated IgG in patients. To provide a rapid and inexpensive alternative that can easily be performed in a standard laboratory or delivered at point-of-care, we adapted our nanobodies to biochemical assays, such as sandwich ELISA or Luminex, to quantify afucosylated IgG1 in patient samples. This contrasts with traditional methods of IgG glycan analysis such as Nano LC–MS, which require purified input material, expensive and highly specialized equipment, and an order of magnitude more time to process samples (Fig. 4*A*). First, we confirmed the specificity of our leading nanobody candidates by immunoprecipitation of IgG from human serum or IgG-depleted serum, demonstrating no binding to other serum glycoproteins (*SI Appendix*, Fig. S7 *A* and *B*). Using serum or purified IgG samples from outpatients from a previously published cohort of convalescent COVID-19 patients (39) whose IgG glycan profiles have been characterized by mass spectrometry, we performed immunoassays capturing human IgG1 (*SI Appendix*, Fig. S8 *A* and *B*), using tetrameric B7 as the detection reagent. Consistent with our studies of homogeneous IgG glycoforms, nanobody-based quantification of afucosylated IgG in both purified patient IgG and serum demonstrated robust correlation with mass spectrometry values (Fig. 4 *B* and *C* and *SI Appendix*, Fig. S9) and using purified IgG or diluted serum had minimal impact on assay output (Fig. 4*D*).

An increase in afucosylated IgG1 has also been observed in SARS-CoV2 infected patients with severe disease (24, 25). To validate these findings and demonstrate the utility of our nanobodies as clinical diagnostics, we used our nanobody-based assay to quantify the levels of afucosylated IgG1 in hospitalized SARS-CoV2-infected patients with moderate or severe COVID-19 as determined by World Health Organization (WHO) criteria (40). Expectedly, patients with moderate to severe disease requiring supplemental oxygen therapy had higher levels of afucosylated IgG1 when compared to patients with moderate disease who did not require supplemental oxygen (Fig. 4*E*).

To demonstrate the use of tetrameric B7 as a rapid clinical prognostic, we performed our nanobody-based assay to quantify afucosylated IgG1 in samples collected from dengue-infected pediatric patients at the time of hospital admission (2–6 d after symptom onset) (21). Using the levels of afucosylated IgG1 derived from the assay, we were able to distinguish patients who would days later develop the mildest form of disease, DF, from those who progressed to DHF or DSS (Fig. 4*F*). Receiver operating characteristic (ROC) analysis of the assay output of both ELISA and Luminex confirmed the prognostic value of IgG glycoform-specific nanobodies in predicting severe dengue disease progression (Fig. 4*G*), comparable to values determined by mass spectroscopy of purified patient IgG (21).

### Nanobodies Can Detect IgG Glycoforms on Live Human Cells.

**Fig. 5.**
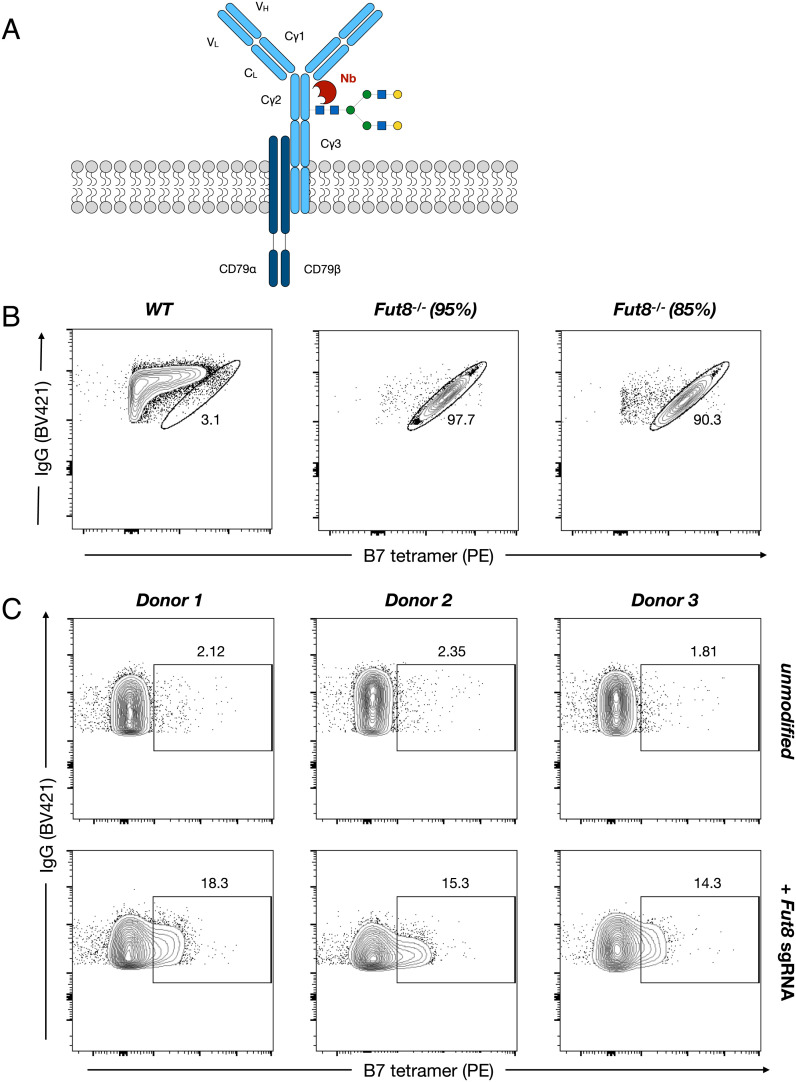
Detection of BCR glycoforms on live human cells. (*A*) Schematic representation of the membrane-bound IgG BCR and its associated glycan. (*B*) Flow cytometry analysis of BCR afucosylation by B7 tetramer staining of IgG1-expressing B-lymphoblastic cell line DB with and without CRISPR/Cas9-mediated *FUT8* knockout. (*C*) Flow cytometry analysis of BCR afucosylation by B7 tetramer staining of primary human IgG^+^ peripheral memory B cells with and without CRISPR/Cas9-mediated FUT8 knockout. CRISPR/Cas9 knockout efficiency was determined by TiDE for all experiments.

Previous approaches characterizing IgG glycosylation have largely focused on secreted antibodies. However, on the surface of B cells, there exists an equivalent membrane-bound form in the BCR, that also harbors the N-linked glycan at Asn297 (Fig. 5*A*). Little is known about the role of this glycan on the BCR, with previous studies suggesting that specific residues, like the core fucose, may be essential for antigen recognition and receptor signaling (41, 42). To determine if our nanobodies could be used to interrogate the BCR glycan structure, we evaluated binding of tetrameric B7 to the IgG1-expressing DB lymphoblastic cell line. On analysis, the BCR of wild-type DB cells was almost uniformly fucosylated. However, CRISPR-mediated knockout of the fucosyltransferase *FUT8* resulted in robust staining, confirming the specificity of our tool for detecting afucosylated IgG BCR (Fig. 5*B*). Notably, the frequency of cells with afucosylated BCR detected by flow-cytometry correlated with the extent of *FUT8* knockout. Next, we extended these methods to primary human class-switched memory B cells derived from peripheral blood. We once again confirmed the specificity of our probes by *FUT8* knockout (Fig. 5*C*). Taken together, these findings demonstrate the capability of these nanobody probes to recognize distinct BCR glycoforms and provide a tool to study glycosylation on living cells.

## Discussion

Historically, antibodies and other glycan-binding reagents have been inadequate for targeting specific glycoproteins. While the field has relied on natural carbohydrate-binding proteins like lectins, their recognition of targets is often nonspecific. Additionally, attempts to generate glycan-binding antibodies have been confounded by the use of heterogeneously glycosylated bait proteins for screening. Here, we exploit the unique structural properties of nanobodies and glycoengineering to generate high-affinity probes that specifically bind to afucosylated and sialylated IgG glycoforms with minimal cross-reactivity. To our knowledge, these probes are first-in-class molecules that selectively bind complex protein glycoforms of a specific glycoprotein. In characterizing these nanobodies, we demonstrate that their binding is dependent on both protein and glycan structures. Furthermore, we show that we can target specific IgG glycoforms both in vitro and in vivo to disrupt protein–protein interactions, highlighting the utility of these reagents beyond glycoform detection (21, 23).

Due to their high affinity and selectivity, these nanobodies were readily adapted to a variety of standard biochemical assays to quantify IgG glycoforms in unpurified patient serum. They accurately reported levels of afucosylated IgG1 in serum from SARS-CoV-2 patients and in the case of dengue, acted as a prognostic to predict whether certain patients progressed to severe disease. While our assay was sufficient in disease contexts where broad global changes in the glycan structure occur, other pathologic conditions with more subtle changes may require reagents with improved affinity or specificity. Additionally, it may be of interest to target other IgG glycan modifications such as galactosylation or bisecting GlcNAc decoration, as the importance of these structures has also been demonstrated in several disease contexts (43). In particular, the sialylated IgG-specific nanobodies described in this study could be applied in the context of autoimmune or inflammatory conditions that have well-documented changes in IgG sialylation (20, 44, 45). Furthermore, while our studies largely focus on lab-based techniques, it may be reasonable to adapt this technology to low-cost clinical platforms that can be deployed at scale.

Finally, despite burgeoning interest in IgG glycosylation, little is known about its regulation or the cells which produce specific glycoforms. For example, it is unknown whether BCR glycoforms are regulated, serve a physiologic role, or match their secreted counterparts. In keeping with this, no studies to date have specifically interrogated the BCR glycan structure. Here, we demonstrate that our nanobodies recognize afucosylated BCR, opening the door for investigation of the complex genetics and cell types that may govern the nebulous processes of glycosylation (46).

## Methods

### Expression and Purification of IgG.

Recombinant antibodies were generated using the Expi293 (ThermoFisher) or Expi293 *FUT8*^*−/*^^*−*^ system using previously described protocols (47). Briefly, an equal ratio of heavy- and light-chain plasmids was complexed with ExpiFectamine in OptiMEM and added to Expi293 cells in culture at 3 × 10^6^ cells/ml. Enhancer 1 and Enhancer 2 were added 20 h after transfection. After 6 d, recombinant IgG antibodies were purified from cell-free supernatants by affinity purification using protein G sepharose beads (GE Healthcare), dialyzed in PBS, filter-sterilized (0.22 μm), concentrated with 100 kDa MWCO spin concentrator (Millipore), purified with Superdex 200 Increase 10/300 GL (GE Healthcare), and finally assessed by SDS–PAGE followed by SafeBlue staining (ThermoFisher). All antibody preparations were more than 95% pure and endotoxin levels were less than 0.05 EU mg^−1^, as measured by the Limulus amebocyte lysate assay. Purified IgG was fluorescently labeled with Alexa Fluor 647-NHS or FITC-NHS (ThermoFisher) at a 15-fold molar excess for 1 h at room temperature and double-dialyzed into PBS.

### Chemoenzymatic Glycoengineering of IgG.

#### LC–ESI–MS analysis of the Fc domains released by IdeS treatment of rituximab derivatives.

LC–ESI–MS analysis was performed with an Exactive Plus Orbitrap Mass Spectrometer (Thermo Scientific) equipped with an Agilent Poroshell 300SB C8 column (5 μm, 1.0 × 75 mm) with a gradient elution of 25−35% aq MeCN containing 0.1% FA for 6 min, 0.4 mL/min. Mass spectra were deconvoluted using MagTran (ver 1.03 b2).

#### Preparation of (Fucα1, 6)GlcNAc-rituximab with immobilized Endo-S2 WT.

Commercial rituximab (22.0 mg, 100 mg/mL, and RefDrug Inc.) was incubated with immobilized (on agarose resin) wild-type Endo-S2 (200:1, wt/wt) at 37°C under gentle shaking for 6 h, when LC–ESI–MS analyses indicated complete cleavage of the N-glycans on the Fc (*SI Appendix*, Fig. S1*A*). The resin was centrifuged, and the deglycosylated antibody was purified by protein A chromatography and exchanged to Tris buffer (100 mM, pH 7.2) to yield (Fucα1,6)GlcNAc-rituximab (20.3 mg, 94%). ESI–MS: calcd for IdeS-treated Fc of (Fucα1,6)GlcNAc-rituximab, M = 24,104 Da; found (*m/z*), 24,102 Da (deconvolution data).

#### Preparation of GlcNAc-rituximab with immobilized Endo-S2 WT and AlfC A-fucosidase in a one-pot manner.

To generate GlcNAc-rituximab, commercial rituximab (Ref Drug Inc., 18.0 mg, and 100 mg/mL) was incubated with immobilized wild-type Endo-S2 following the procedure above. After the Fc glycan was completely removed, α-fucosidase AlfC from Lactobacillus casei (50:1, wt/wt) was added to the mixture and incubated at 37°C for 16 h, when LC–MS analyses indicated complete cleavage of the core fucose on the Fc (*SI Appendix*, Fig. S1*B*). The resin was centrifuged down, and the antibody was isolated and purified by protein A chromatography, exchanged to Tris buffer (100 mM, pH 7.2) to yield GlcNAc-rituximab (15.2 mg, 86%). ESI–MS: calcd for IdeS-treated Fc of GlcNAc-rituximab, M = 23,958 Da; found (*m/z*), 23,956 Da (deconvolution data).

#### Enzymatic transglycosylation of (Fucα1, 6)GlcNAc-rituximab or GlcNAc-rituximab to generate rituximab glycoforms.

A solution of (Fucα1,6)GlcNAc-rituximab (9.0 mg) or GlcNAc-rituximab (9.0 mg) in a Tris buffer (100 mM, pH 7.2, final antibody concentration 15 mg/mL) and G2-glycan oxazoline (30 eq) was incubated with Endo-S2 D184M mutant (0.05 mg/mL) at 30°C for 15 min. LC–MS analyses (*SI Appendix*, Fig. S1 *C* and *D*) indicated the complete transglycosylation. The mixture was purified by protein A chromatography and exchanged to PBS buffer (100 mM, pH 7.4) to yield G2F-rituximab (8.1 mg, 88%) or G2-rituximab (8.3 mg, 90%). ESI–MS: calcd for IdeS-treated Fc of G2F-rituximab, M = 25,523 Da; found (*m/z*), 25,522 Da (deconvolution data); calcd for IdeS-treated Fc of G2-rituximab, M = 25,377 Da; found (*m/z*), 25,376 Da (deconvolution data). The homogeneity of glycoengineered rituximab was further confirmed by fluorescence labeling of released N-glycans with 2-AA (*SI Appendix*, Fig. S2 *B* and *C*), compared to the heterogeneity of glycans for native Rituxan (*SI Appendix*, Fig. S2*A*). The identity of the 2-AA labeled released glycans was confirmed by MALDI-TOF-MS analysis (*SI Appendix*, Fig. S2 *D–F*).

### Identification of IgG Fc Glycoform-Specific Nanobodies.

We used a previously published yeast surface display library (>5 × 10^8^ variants) that recapitulates the native llama VHH repertoire (30). The library displays an HA-tagged nanobody at the terminus of a synthetic stalk sequence, whose expression is controlled by an inducible Gal promoter. In the presence of galactose, 12 to 18% of the naïve library typically expresses the nanobody protein.

For round 1, 5 × 10^9^ yeast (10 × expected diversity) were induced for 48 h in YEP-galactose tryptophan dropout (-Trp) medium and washed in staining buffer (20 mM HEPES, pH 7.5, 150 mM sodium chloride, 0.1% (w/v) bovine serum albumin). For negative selection, yeast was resuspended in 5 mL staining buffer containing 500 nM rituximab-G2F-Alexa Fluor 647. Yeast was incubated for 1 h at 4°C, washed in cold staining buffer, and resuspended in 4.5 mL staining buffer with 500 µL anti-Alexa Fluor 647 microbeads (Miltenyi). Yeast was incubated with microbeads for 20 min at 4°C, washed in cold staining buffer, and depleted of G2F-binders on a MACS LS column (Miltenyi). For positive selection, yeast was resuspended in 5 mL staining buffer with 500 nM rituximab-G2-FITC or rituximab-S2G2F-FITC. Yeast was incubated for 1 h at 4°C, washed in cold staining buffer, and resuspended in 4.5 mL staining buffer with 500 µL anti-FITC microbeads. Yeast was incubated with microbeads for 20 min at 4°C, washed in cold staining buffer, and G2- or S2G2F-binders were captured on a MACS LS column and recovered in a YEP-glucose (-Trp) medium.

For round 2 of selection, for 1.5 × 10^8^ induced yeast, the procedure outlined in round 1 was performed with the fluorophores swapped (i.e., rituximab-G2F-FITC and rituximab-G2-Alexa647 or rituximab-S2G2F-Alexa Fluor 647). For rounds 3–5, FACS was used in place of MACS. For round 3, 1.5 × 10^7^ induced yeast were stained with 500 nM rituximab-G2F-Alexa Fluor 647 and 250 nM rituximab-G2-FITC or rituximab-S2G2F-FITC. FITC^+^Alexa Fluor 647^−^ clones were sorted into YEP-glucose (-Trp) and expanded. For round 4, 1.5 × 10^7^ induced yeast were stained with 500 nM rituximab-G2F-FITC and 250 nM rituximab-G2-Alexa Fluor 647 or 250 nM rituximab-S2G2F-Alexa647. FITC^−^Alexa647^+^ clones were sorted into YEP-glucose (-Trp) and expanded. For round 5, 1.5 × 10^7^ induced yeast were stained with 500 nM rituximab-G2F-Alexa Fluor 647 and 100 nM rituximab-G2-FITC or 100 nM rituximab-S2G2F-FITC. FITC^+^Alexa Fluor 647^−^ clones were sorted into YEP-glucose (-Trp) and expanded.

8 × 10^6^ yeast were spun down and resuspended in 30 µL 0.2% sodium dodecyl sulfate (v/v) and heated at 94°C for 4 min to lyse yeast. Yeast was spun down at 10,000 × *g*, and 1 µL of supernatant was used as a template for a PCR reaction using [primer3, primer4]. Next-generation sequencing of post-round 5 nanobody sequences was performed by a MiSeq Nano (Illumina) with 10% PhiX to yield dominant clones (G2: C11 and D3) and (S2G2F: H9 and C5).

### Expression and Purification of Nanobodies.

Nanobodies were expressed and purified similarly to previously reported methods (30, 35, 48). Nanobody sequences were amplified with [primer 5, primer 6] and cloned into pET26-b(+) expression vector with His tag and AviTag using Gibson Assembly (NEB) and transformed into BL21(DE3) *Escherichia coli* (NEB). Nanobody multimers were generated using multipart Gibson Assembly with unique linker regions to preserve correct orientation. Bacteria were grown in terrific broth at 37ºC overnight, and the next day a 1:100 culture was grown until an OD of 0.7–0.9, when 1 mM IPTG was added. After 20–24 h of shaking at 25ºC, *E. coli* were pelleted and resuspended in SET buffer (200 mM Tris, pH 8.0, 500 mM sucrose, 0.5 mM EDTA, and 1 × complete protease inhibitor (Sigma)) and rocked for 30 min at room temperature, followed by the addition of 2 × volume of deionized water and 45 min more rocking. NaCl was added to 150 mM, MgCl_2_ to 2 mM, and imidazole to 20 mM before pelleting cell debris at 17,000 × *g* for 20 min. The periplasmic fraction was filtered with a 0.22 um filter and incubated with 4 mL 50% Ni-NTA resin equilibrated in wash buffer (20 mM HEPES, pH 7.5, 150 mM NaCl, and 40 mM imidazole) (Qiagen) per liter of initial bacterial culture. The supernatant and resin were rocked for 1 h at room temperature and then pelleted at 50 × *g* for 1 min. Resin was washed on a column with 10 volumes of wash buffer before elution with elution buffer (20 mM HEPES, pH 7.5, 150 mM NaCl, and 250 mM imidazole). Eluted protein was concentrated with 3 kDa MWCO filters (Amicon) before size-exclusion chromatography (GE Healthcare). Proteins were stable at 4ºC.

For tetramerization, nanobody monomers were biotinylated in vitro with BirA (Avidity) for 1 h at room temperature according to the manufacturer’s directions, double-desalted using Zeba Spin Desalting columns 7K MWCO (ThermoFisher), and purified by size-exclusion chromatography. For in vivo biotinylation, CVB-T7 POL *E. coli* (Avidity) were used to express nanobodies, and at the time of induction, 50 µM D-biotin was added to the culture. Streptavidin conjugates were complexed in a 1:4 ratio with biotinylated monomers by adding 1/4th volume of conjugate every 10 min for a total of 40 min.

For nanobody-Fc fusions, nanobody sequences were directly fused to human IgG1 Fc residues 216–447. Importantly, Asn297 was mutated to alanine to prevent nanobody binding. These constructs were expressed and purified in the same manner as antibodies (see *Expression and Purification of IgG*).

### Surface Plasmon Resonance.

SPR was performed on a Biacore T200 machine (Cytiva Life Sciences). In some experiments, purified IgG glycoforms diluted in HBS-EP^+^ were immobilized on the surface of a Protein A or Protein G CM5 sensor chip at 1,000 RU (~50 nM). Purified nanobodies were flowed over IgG-bound sensor chips at the indicated concentrations at 30 µL/min for 60 s, followed by 600 s of dissociation. Sensor chips were regenerated with 10 mM Glycine-HCl pH 1.5.

In other experiments, purified His-tagged nanobodies were immobilized on the Ni^2+^-activated surface of NTA sensor chips at 500 RU (50 nM). Purified IgG was flowed over nanobody-bound sensor chips at the indicated concentrations at 30 µL/min for 60 s, followed by 600 s of dissociation. Sensor chips were regenerated with 350 mM EDTA.

All kinetic constants were calculated using GraphPad Prism v9. For nanobody monomer binding, sensorgrams were fit using a 1:1 Langmuir binding model, and kinetic constants were reported. For tetramer binding, the association phase was fit separately using an association kinetics model simultaneously fitting the association rate constant for each concentration. The dissociation phase was fit to a biexponential decay model with two dissociation rate constants (one fast and one slow) shared between each concentration.

For epitope mapping experiments, rituximab G2 diluted in HBS-EP^+^ was immobilized on the surface of a Protein A or Protein G CM5 sensor chip at 1,000 RU (~50 nM). Purified B7 or FcγRIIIA was injected at 10s at 30 µL/min for 100 s to achieve saturation. Immediately after that, a mixture of purified B7 and FcγRIIIA at the same concentration as in the primary injection was injected at 30 µL/min for 100 s, followed by dissociation.

### Affinity Maturation of C11.

Using degenerate NNK oligos, assembly PCR was used to generate a site saturation mutagenesis library of C11, where one codon in each CDR was mutated at a time, for a total of 0–3 amino acid CDR mutations per nanobody clone (Fig. 2*A*). The pooled assembly PCR reaction was amplified so that its ends overlapped with the surface display vector used in the initial rounds of selection. Vector and insert DNA were electroporated into *Saccharomyces cerevisiae* strain BJ5465 (ATCC 208289) to generate a library of 1.4 × 10^7^ transformants, which were plated on YEP-glucose (-Trp) agar. Plates were scraped, and 1.4 × 10^8^ yeast were induced in YEP-galactose (-Trp) for 48 h. For round 1, yeast was washed in staining buffer and costained with 125 nM rituximab-G2F-FITC and 2.5 nM rituximab-G2-Alexa Fluor 647 (50-fold excess G2F). FITC^-^Alexa Fluor 647^+^ clones were sorted into YEP-glucose (-Trp), expanded, and induced for round 2. Clones were induced and co-stained with 37.5 nM rituximab-G2F-Alexa647 and 750 pM rituximab-G2-FITC (50-fold excess G2F). FITC^+^Alexa Fluor 647^–^ clones were sorted and plated onto YEP-glucose (-Trp) agar. Then, 288 individual clones were induced in duplicate 96-well plates and stained with 200 pM rituximab-G2-Alexa Fluor 647 or 10 nM rituximab-G2F-Alexa Fluor 647. Highly selective clones were selected and sequenced for further experiments.

### Nanobody ELISA.

For some experiments, half-well 96-well plates were coated with 30 µL of 10 µg/mL mouse anti-IgG1 (ThermoFisher) overnight. Plates were washed with PBST (0.05% Tween-20) 3 times, blocked with 2% BSA in PBS for 1 h at room temperature, washed, incubated with recombinant IgG, purified patient IgG, or patient serum, washed, incubated with nanobody-streptavidin-HRP conjugates (1:1,000, BioLegend), washed, developed with a TMB substrate, quenched with 1 M phosphoric acid, and read at 450 nm on a spectrophotometer.

For other experiments, half-well 96-well plates were coated with 30 µL of 10 µg/mL nanobody overnight. Plates were washed with PBST (0.05% Tween-20) three times, blocked with 2% BSA in PBS for 1 h at room temperature, washed, incubated with recombinant IgG, purified patient IgG, or patient serum, washed, incubated with anti-human IgG–HRP conjugates (1:5,000, JacksonImmunoResearch), washed, developed with a TMB substrate, quenched with 1M phosphoric acid, and read at 450 nm on a spectrophotometer.

### Nanobody Luminex.

Magplex microspheres (region 45) were conjugated to mouse anti-human IgG1 (ThermoFisher) using xMAP Ab Coupling kit, as per the manufacturer’s instructions, and blocked with 1% BSA in PBS overnight. Then, 50 µL microspheres and 50 µL diluted recombinant IgG, purified patient IgG, or patient serum were shaken at 500 rpm in a 96-well plate for 1 h. Microspheres were washed 3 times with 1% BSA in PBS and shaken with nanobody-streptavidin-PE conjugates for 30 min. Microspheres were washed, and median fluorescent intensities were calculated using Luminex 200 Instrument System (ThermoFisher).

For other experiments, Magplex microspheres (region 45) were conjugated to S2G2F-specific clone H9 (10 µg/10^6^ beads), as per the manufacturer’s instructions, and blocked with 1% BSA overnight. Then, 50 µL microspheres and 50 µL diluted recombinant IgG were shaken at 500 rpm in a 96-well plate for 1 h. Microspheres were washed 3 times with 1% BSA in PBS and shaken with R-PE-conjugated Fab_2_ goat antihuman IgG Fc (Jackson Immunoresearch) for 30 min. Microspheres were washed, and median fluorescent intensities were calculated using Luminex 200 Instrument System (ThermoFisher).

### ELISA-Based FcγR Binding Assay.

Recombinant FcγR ectodomains were expressed in Expi-293F and purified with Ni-NTA resin as in previously described protocols (47). High-binding 96-well microtiter plates (Nunc) were incubated with 10 µg/mL recombinant FcγRI or FcγRIIIA(V) overnight at 4°C. Plates were then blocked with PBS plus 2% (w/v) BSA. IgG immune complexes were prepared by incubation of an anti-NP (4-hydroxy-3-nitrophenylacetyl) antibody 3B62 with NP-BSA (27 conjugations) at a 10:1 molar ratio for 1 h at 4°C. Nanobodies were serially diluted 1:3 in PBS, with a starting concentration of 19.2 nM. IgG immune complexes or monomeric 3B62 were brought to a concentration of 20 µg/mL or 2 µg/mL, respectively, and precomplexed at a 1:1 (v/v) ratio for 1h at room temperature and then captured on FcγR-coated plates. Following 1h incubation, bound IgG was detected using a 1:5,000 dilution of horseradish peroxidase (HRP)-conjugated goat F(ab’)_2_ anti-human IgG (H + L) (Jackson Immunoresearch). Plates were developed with a TMB (3,3′,5,5′-tetramethylbenzidine) two-component peroxidase substrate kit. Reactions were quenched with 1M phosphoric acid. Absorbance at 450 nm was recorded using a SpectraMax Plus spectrophotometer (Molecular Devices). Background absorbance was subtracted for samples, and % maximum binding was determined against an IgG or immune complex-only control.

### IgG Fc Glycan and IgG Subclass Analysis.

The subclass distribution and Fc glycan composition of IgGs were determined by mass spectrometry at the Institute of Biotechnology of the Cornell University, as described previously (21, 23). Briefly, IgGs were purified from plasma or serum samples by protein G purification and dialyzed against PBS. Assay reproducibility was determined by assessing the Fc glycan profile from three subjects in two independent experiments. Research personnel involved in Fc glycan analysis had no access to clinical information and characteristics of the patient samples.

### Glycan Array.

N-glycan arrays (Z-Biotech) were used according to the manufacturer’s instructions. Briefly, slides were blocked with Glycan Array Blocking Buffer for an hour on a shaker at 85 rpm. After an hour, the blocking buffer was removed and 200 µL B7 (0.5 mg/mL or 0.05 mg/mL) or biotinylated-AAL (10 µg/mL) was added. Slides were incubated for 2 h under shaking at 200 rpm and then washed three times with Wash Buffer (50mM Tris-HCl, 137 mM NaCl, 0.05% Tween 20, and pH 7.6). Then, 200 µL of 1 µg/mL Streptavidin-Cy3 (Vector labs) was added for 1 h under shaking at 85 rpm. Slides were washed three times with Wash Buffer, dried, and then scanned with a Typhoon FLA-9500 scanner (GE Healthcare).

### Immunoprecipitation.

Streptavidin-coated Dynabeads (ThermoFisher) were incubated with 5:1 molar excess biotinylated nanobody for 1 h at room temperature and then washed three times with PBS. Dynabeads were incubated with serum or IgG-depleted serum for 1 h, after which they were washed 3 times with PBS. Beads were boiled for 5 min at 95°C in SDS loading buffer and loaded onto a 4 to 12% Bis-Tris protein gel for analysis.

### Generation of FUT8 Knockout Expi-293F and DB Cell Lines.

CRISPR–Cas9 guide RNAs targeting human *FUT8* were assembled with Cas9-3NLS nuclease (Synthego) via incubation at 37 °C for 15 min. Cas9/RNP complexes were nucleofected into 2 × 10^6^ cells using the SF Cell Line 4D-Nucleofector kit according to the manufacturer’s instructions (Lonza). After a week of culture, indel frequencies were quantified using TIDE software as described previously (49). Sequence for the single-guide RNA (sgRNA) molecule used is as follows: ACAGCCAAGGGTAAATATGG.

### B Cell Depletion Model.

All in vivo experiments were performed in compliance with federal laws and institutional guidelines and have been approved by the Rockefeller University Institutional Animal Care and Use Committee. Mice were bred and maintained at the Comparative Bioscience Center at the Rockefeller University. For all experiments, huCD20 transgenic mice on an FcγR-humanized background (34) (males and females; 8–12 wk old) were administered 0.5 mg/kg rituximab G2 (anti-huCD20) to deplete B cells. For nanobody prophylaxis, nanobody-Fc fusion proteins were administered (2.5 mg/kg) together with rituximab. For nanobody treatment, nanobody-Fc fusion proteins were administered (2.5 mg/kg) 2 h after rituximab. Mice were bled on Days 0, 1, and 2 for analysis. Whole blood was lysed (RBC lysis buffer; BioLegend) for 5 min at room temperature and resuspended in FACS buffer (PBS containing 1% w/v BSA and 2 mM EDTA). Cells were labeled with fluorescently conjugated antibodies anti-CD45 (PE-Cy7) and anti-huCD20 (APC), as well as 7-AAD viability dye (ThermoFisher). Samples were collected on an Attune NxT flow cytometer (ThermoFisher), and B cell frequencies (B220^+^ in CD45^+^) were calculated using FlowJo (v10.6).

### Surface BCR Analysis.

The B lymphoblast cell line, DB (ATCC), was utilized for evaluating nanobody binding to surface BCR. Cells were stained in FACS buffer and labeled with fluorescently conjugated anti-hIgG Fc (BV421) at 1:200 (BioLegend), B7-tetramer (PE) at 1 µg/ml, and 7-AAD viability dye (ThermoFisher) at 1:1,000.

For analysis of primary human B cells, buffy coats were obtained from the New York Blood Center. Samples were diluted in RPMI-1640, and peripheral blood mononuclear cells were separated by Ficoll gradient centrifugation. Class-switched B cells were enriched by MACS using a class-switched memory B cell enrichment kit (Miltenyi). B cells were cultured for 24 h in B cell media (RPMI-1640 supplemented with 10% fetal bovine serum, 55 µM β-mercaptoethanol, 2 mM L-glutamine, 1 mM sodium pyruvate, penicillin/streptomycin (1×), 10 mM HEPES, and 2 µg/ml anti-RP105 (clone MHR73-11, BioLegend)). CRISPR–Cas9 guide RNAs targeting human FUT8 were assembled with Cas9-3NLS nuclease (Synthego) via incubation at 37°C for 15 min. Cas9/RNP complexes were nucleofected into 1 × 10^6^ cells using the P3 Primary Cell 4D-Nucleofector kit and program EH-140 (Lonza). After 48 h, indel frequencies were quantified using TIDE software as described previously. The sequence for the single-guide RNA (sgRNA) molecule used is as follows: ACAGCCAAGGGTAAATATGG. Cells were stained as described for the DB cell line. Samples were collected on an Attune NxT flow cytometer, and data were analyzed with FlowJo.

### Patient Samples.

For serum or purified IgG in Fig. 4 *A–C*, samples were obtained from a previously described patient cohort of convalescent COVID-19 patients (39). For dengue virus-infected patients in Fig. 4 *E* and *F*, purified IgG from a previously published dengue virus-infected cohort was used (21). All samples were deidentified and obtained under approval from the Rockefeller University Institutional Review Board.

Sera from adult patients (≥21 y old) with COVID-19, presenting to the Emergency Room of Weiler Hospital, the Einstein Campus of Montefiore Medical Center (MMC) in the Bronx, New York, were collected between September 2020 and May 2021. Inclusion criteria were signs and symptoms associated with COVID-19, documented new SARS-CoV-2 infection, diagnosed by real-time reverse transcription PCR (RT-PCR) of nasopharyngeal secretions (obtained via deep nasopharyngeal swabs), and admission to the hospital. Exclusion criteria were age <21, history of prior SARS-CoV-2 infection, or no COVID-19-associated symptoms. Patients were categorized by WHO score (40). Hospitalized patients were grouped into those with moderate disease but not requiring supplemental oxygen therapy (WHO score 4; n = 13) and those with moderate–severe disease (WHO scores 5–9; n = 18) requiring supplemental oxygen by nasal canular (WHO 5), high flow, Bilevel Positive Airway Pressure (BiPAP), and/or mechanical ventilation (WHO 6–9). There were no significant differences in demographics between the groups. Patients with WHO score 4 had a mean age of 64 ± 15 y, and 8/13 (61.5%) were male; patients with WHO score 5–9 had a mean age of 66 ± 12 y, and 9/18 (50%) were male. Leftover sera (from clinical lab evaluations) were stored at 4°C in the clinical hematology lab for 24–48 h, collected, coded without identifiers, aliquoted, and stored at −80°C on the day obtained until tested. The study was approved by the Albert Einstein College of Medicine and MMC Institutional Review Board as Category 5—Research involving materials (data, documents, records, and or specimens) that have been collected or will be collected solely for nonresearch purposes (such as medical treatment or diagnosis). Because we only collected left-over samples from routine clinical evaluations, no individual consent was required. All samples were coded/deidentified, and all identifiers are destroyed after the completion of medical record reviews and studies.

### Statistics.

An unpaired two-tailed *t* test was used when comparing two groups. One-way ANOVA with Bonferroni’s post hoc test was used when comparing more than two groups. GraphPad Prism software (v9.1) was used for all statistical analyses. *P* values of ≤0.05 were considered statistically significant.

## Supplementary Material

Appendix 01 (PDF)

## Data Availability

All study data are included in the article and/or *SI Appendix*.
