# Supplementary material for: Synthetic nanobodies as tools to distinguish IgG Fc glycoforms
Source: Proc Natl Acad Sci U S A. 2022 Nov 21;119(48):e2212658119. doi: 10.1073/pnas.2212658119 (PMC9860306; doi:10.1073/pnas.2212658119)
Supplement: Supplementary file 1 — Appendix 01 (PDF) [file pnas.2212658119.sapp.pdf]

Supporting Information for

Synthetic nanobodies as tools to distinguish IgG Fc glycoforms

Kevin Kao[1,5], Aaron Gupta[1,5], Guanghui Zong[2], Chao Li[2], Isabell Kerschbaumer[3], Sara Borghi[1] Jacqueline M Achkar[3,4], Stylianos Bournazos[1], Lai-Xi Wang[2], Jeffrey V. Ravetch[1]*

Jeffrey V. Ravetch[1]
Email: ravetch@rockefeller.edu

This PDF file includes:

Supporting text
Figures S1 to S9

Other supporting materials for this manuscript include the following:

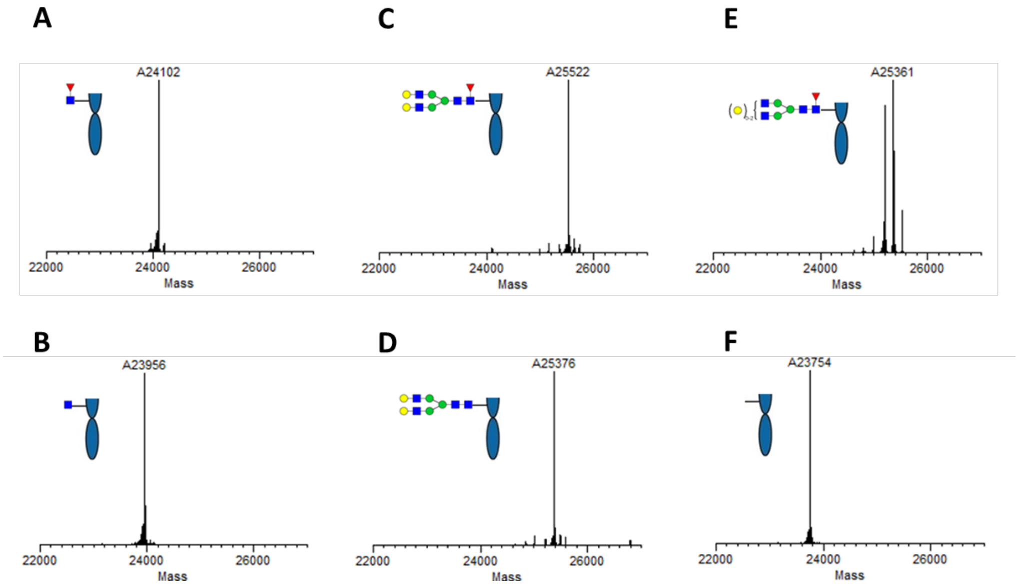

Fig. S1. LC-ESI-MS analysis of the Fc domains released by IdeS treatment of the glycan remodeled and commercial Rituximab (**A**) (Fucα1,6) GlcNAc-Rituximab; (**B**) GlcNAc-Rituximab; (**C**) G2F-Rituximab; (**D**) G2-Rituximab; (**E**) commercial Rituximab; (**F**) The Fc domain of commercial Rituximab after PNGase F catalyzed deglycosylation.

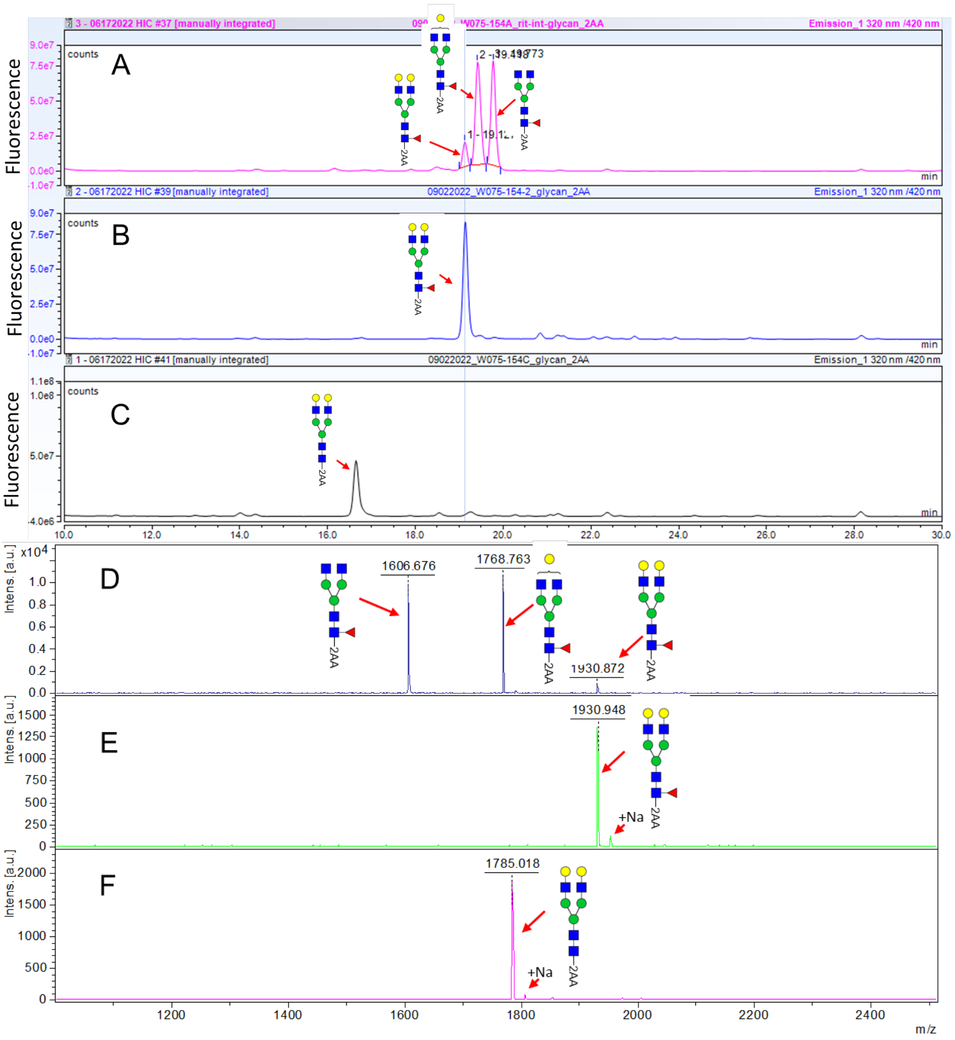

Fig. S2. Fluorescent HPLC profiles and MALDI-TOF MS analysis of 2-aminobenzoic acid (2-AA) labeled Fc glycans released from different Rituximab glycoforms. **(A)** HPLC analysis of the 2-AA labeled Fc glycan from commercial Rituximab; **(B)** HPLC analysis of the 2-AA labeled Fc glycan from Rituximab Fc glycoengineered with G2F; **(C)** HPLC analysis of the 2-AA labeled Fc glycan from Rituximab Fc glycoengineered with G2; **(D)** MALDI-TOF-MS analysis of the 2-AA labeled Fc glycan from commercial Rituximab Fc; **(E)** MALDI-TOF-MS analysis of the 2-AA labeled Fc glycan from the Rituximab glycoengineered with G2F; **(F)** MALDI-TOF-MS analysis of the 2-AA labeled Fc glycan from the Rituximab glycoengineered with G2. For 2-AA glycan labeling, the Fc N-glycans were released from the rituximab by PNGase F treatment and were subsequently labeled by reductive amination with a-aminobenzoic acid, following the manufacturer's protocol (Sigma-Aldrich GlycoProfile^{TM} 2-AA Labeling Kit). Analytical RP-HPLC was performed on a Thermo Scientific^{TM} Vanquish^{TM} UHPLC system equipped with a fluorescence detector F (excitation, 320 nm; emission, 420 nm). Separations were performed using a C18 column (Thermo Scientific Accucore^{TM}, 3 × 150 mm, 2.6 μm) at a flow rate of 0.6 mL/min using a linear gradient of 0-15% MeCN containing 0.1% TFA over 30 min at 30 °C. Fluorescent HPLC analysis and quantification indicated that the Fc N-glycans from the commercial Rituximab consisted of three forms, G2F, G1F, and G0F, which were present in the ratios of 9.3 : 47.7 : 43.0. The glycoengineered Rituximab glycoforms G2F and G2 showed a single homogeneous N-glycan (G2F and G2 respectively), without detection of other N-glycans.

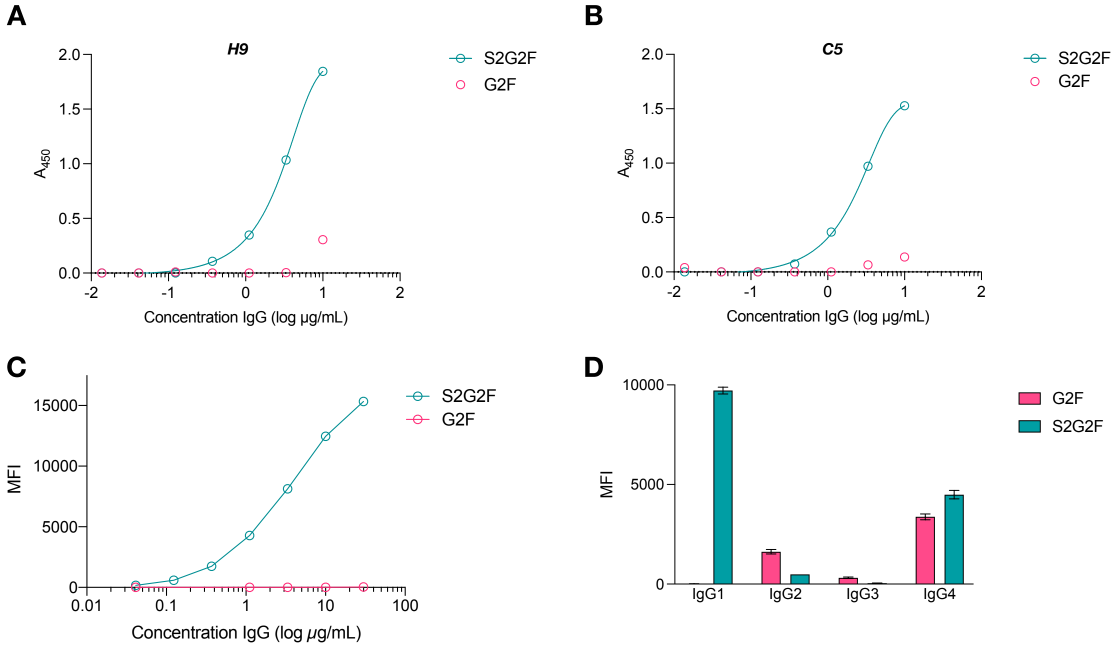

Fig. S3. Specificity of sialylated IgG-specific nanobodies. (A and **B**) Sandwich ELISA demonstrating specific nanobody capture of Rituximab S2G2F by clones H9 and C5. **(C)** Bead-based (Luminex) capture of IgG glycoforms by clone H9. **(D)** Bead-based (Luminex) capture of G2F and S2G2F glycoforms of all IgG subclasses. Data displayed as mean ± SEM (n = 3). Data in **(A-C)** were fitted by nonlinear regression analysis.

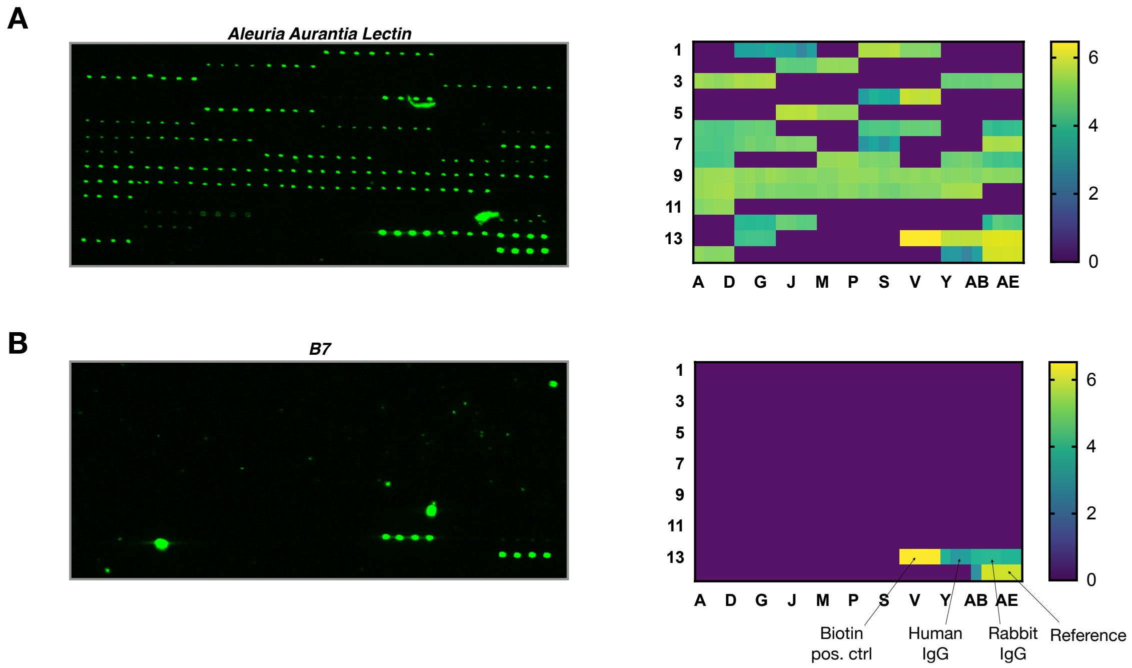

Fig. S4. Clone B7 does not bind N-linked glycans without the IgG protein backbone. (A and **B)** Fluorescent signal (Cy3) of Aleuria Aurantia Lectin (AAL) or B7 representing binding to an array of immobilized N-linked glycans. Each condition is provided as a technical quadruplicate oriented horizontally. Conversion of median fluorescent intensity (MFI) to representative heatmap. MFI scale is given as $\log_2$. Immobilized biotin is a positive control for streptavidin-Cy3 binding.

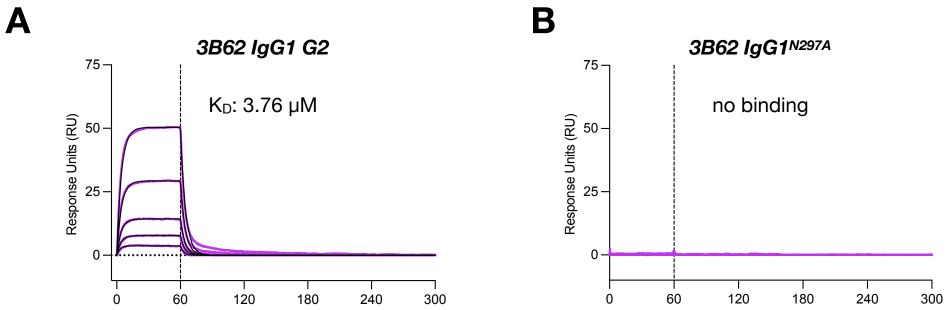

Fig. S5. Clone B7 does not bind aglycosylated IgG. (A and **B)** Binding kinetics of B7 with anti-NP clone 3B62 IgG1 G2 and its aglycosylated 3B62 N297A mutant. Purple traces are raw data, while 1:1 Langmuir global kinetic fits are shown in black. Sample concentrations began at 256 nM with 2-fold serial titration until 16 nM.

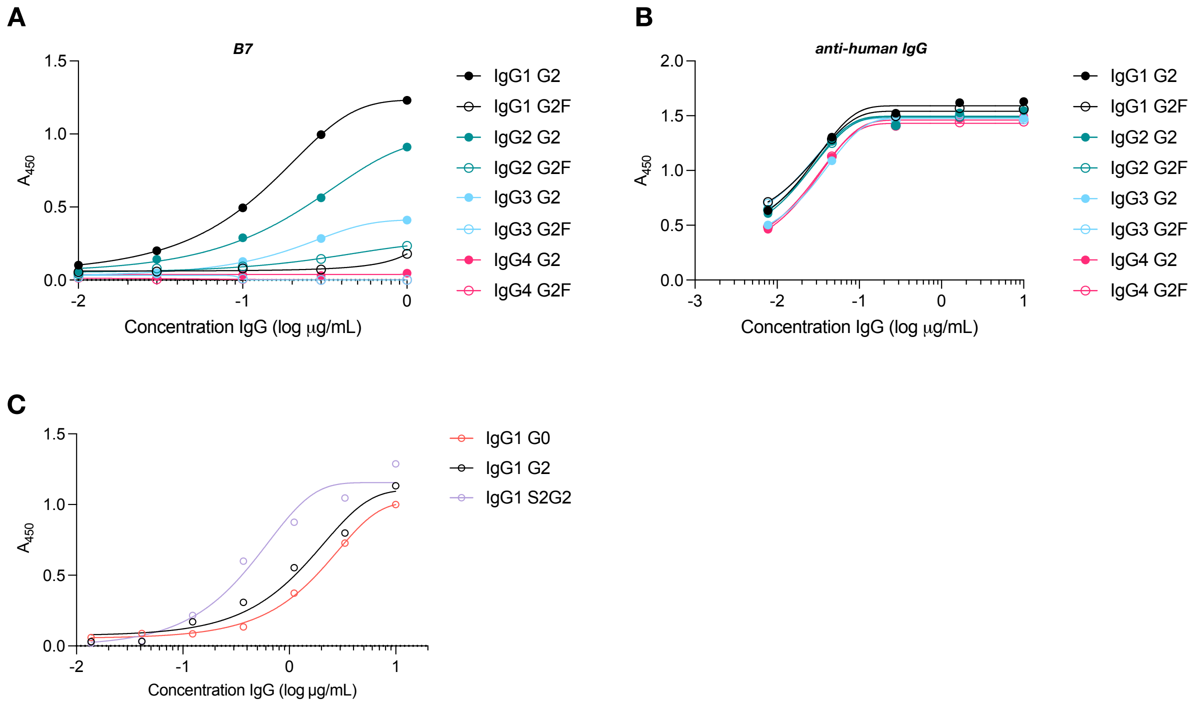

Fig. S6. IgG subclass and glycoform specificity of clone B7. (A) Sandwich ELISA evaluating subclass and glycoform specificity of clone B7. Subclass specificity is IgG1 > IgG2 > IgG3 >> IgG4. Binding to fucosylated IgGs is minimal. **(B)** The human IgG detection reagent in **A** does not have preference for subclass or glycoform of IgG. **(C)** B7 retains binding to all major afucosylated glycoforms present in human serum. Data in **(A-C)** were fitted by nonlinear regression analysis.

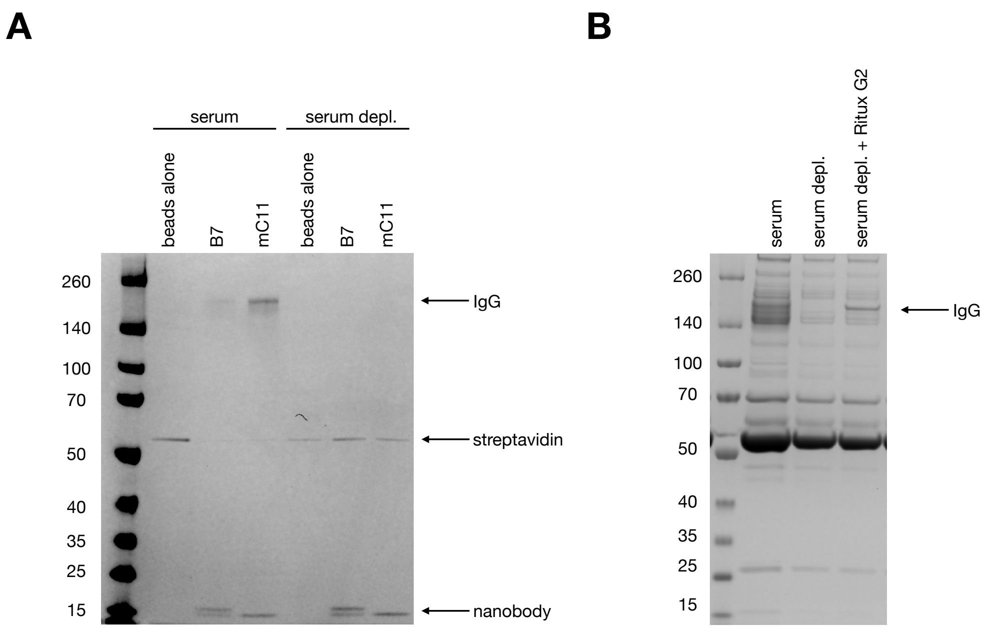

Fig. S7. Immunoprecipitation of IgG from human serum by glycoform-specific nanobodies. (A) SDS-PAGE comparing B7 and mC11 immunoprecipitation of IgG from intact (left three lanes) or IgG-depleted human serum (right three lanes). **(B)** Coomassie gel of intact, IgG-depleted, and IgG-depleted serum reconstituted with Rituximab G2 confirming appropriate depletion or reconstitution of IgG.

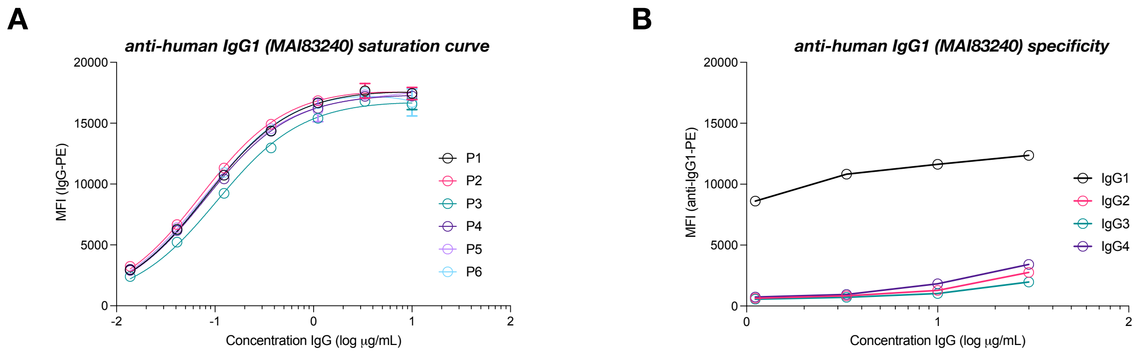

Fig. S8. Capture of IgG1 by anti-human IgG1 clone MAI-83240. (A) Luminex quantification of capture of purified patient IgG by beads coated with clone MAI-83240. Data was fitted by nonlinear regression analysis. **(B)** Subclass specificity of clone MAI-83240.

A

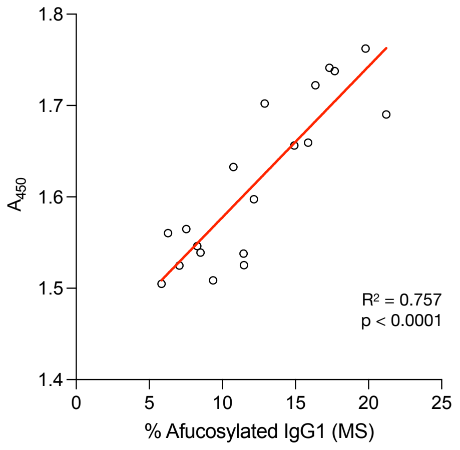

Fig. S9. ELISA based quantification of afucosylated IgG levels in patient serum. (A) Sandwich ELISA demonstrating strong correlation of OD450 with mass spectrometry determined levels of afucosylated IgG. Statistics determined by Pearson correlation analysis.
